# Predicting the efficacy of opioid sequestration by intravenous lipid emulsion using biologically relevant in vitro models of drug distribution

**DOI:** 10.1038/s41598-022-21790-4

**Published:** 2022-11-04

**Authors:** Marta Tikhomirov, Paweł Jajor, Tomasz Śniegocki, Błażej Poźniak

**Affiliations:** 1 Faculty of Veterinary Medicine, Department of Pharmacology and Toxicology, Wroclaw University of Environmental and Life Sciences, 50-375 Wrocław, Poland; 2 Department of Pharmacology and Toxicology, National Veterinary Research Institute, 24-100 Puławy, Poland

**Keywords:** Experimental models of disease, Preclinical research

## Abstract

Intravenous lipid emulsions (ILE), among other uses, are utilized in the treatment of poisonings caused by lipophilic substances. The body of evidence regarding the benefits of this treatment is growing but information about opioids-ILE interaction is still very scarce. In this work, the impact of ILE on the distribution of buprenorphine, fentanyl and butorphanol used in various concentrations (100–500 ng/ml) was investigated. Two different in vitro models were used: disposition of the drugs in plasma after ultracentrifugation and distribution into the simulated biophase (cell monolayer of 3T3 fibroblasts or J774.E macrophages). We confirmed the ability of ILE to sequester the three drugs of interest which results in their decrease in the aqueous part of the plasma by 34.2–38.2%, 11.7–28.5% and 6.0–15.5% for buprenorphine, fentanyl and butorphanol, respectively. Moreover, ILE affected the drug distribution to the biophase in vitro, however, in this case the drug concentration in cells decreased by 97.3 ± 3.1%, 28.6 ± 5.4% and 13.0 ± 7.5% for buprenorphine, fentanyl and butorphanol, respectively. The two models revealed notable differences in ILE’s potential for drug sequestration, especially for buprenorphine. Similar, but not as pronounced tendencies were observed for the two other drugs. These discrepancies may result from the difference in protein abundance and resulting drug-protein binding in both systems. Nevertheless, the results obtained with both in vitro models correlated well with the partition coefficient (logP) values for these drugs.

## Introduction

Intravenous lipid emulsions (ILE) have an established position in the management of local anesthetic toxicity^1–4^. After the success in this field, ILE was used in clinical conditions for multiple types of overdoses^5^ and randomized clinical trials have demonstrated a benefit of ILE in multiple toxicities^6–9^. Numerous reports investigate the potential use of ILE in overdoses of such miscellaneous substances as propranolol^10^, cocaine^11^, diltiazem^12^ or caffeine^13^, to name just a few. The majority of published works tend to test the lipid rescue therapy in a clinical setting on animals or humans^5–7,14,15^. Under clinical conditions, the results of such an intervention shed light on the true therapeutic relevance and all sources of variability and mechanisms involved are operative. Consequently, in vivo studies are indispensable to assess its efficacy. However, it is known that not all drug poisonings respond well to ILE treatment^5,9,16,17^ and the trial-and-error attempt seems risky and inefficient. The enormous number of molecules that may interact with ILE giving a potentially beneficial outcome in rescue attempts requires a more high-throughput approach. That is why in vitro models can be especially useful in the predictions of ILE efficacy.

One of the earliest proposed, and still valid today, mechanisms of ILE action is the so-called “lipid sink” complemented with “lipid shuttle”^4,18,19^. Based on the ability to sequester the toxicant in the lipid droplets and transport them to the less vulnerable organs, most in vitro works concentrate their efforts on the investigation of the drug’s lipophilicity^20–22^. In the classical drug development studies, the lipophilicity of the active compound is reported as the partition coefficient^23^. This parameter, typically measured between n-octanol and water, provides information about the drug’s ratio of concentrations measured between the two immiscible solvents under equilibrium conditions^23^. Nevertheless, this simple test does not reflect the conditions and factors present in the living organism. Therefore, some in vitro tests in this field tend to explore the compound’s lipophilicity, but under more biologically relevant conditions^20,24–26^.

In our work, we investigated the interactions between ILE and three selected opioids, namely buprenorphine (BPN), fentanyl (FTL) and butorphanol (BTL). The selection of opioids was based on the relatively high lipophilicity of some drugs belonging to this class^27–29^ as well as high prevalence and serious societal consequences of overdose cases related to these drugs^30^. Even though opioid receptors’ antagonists, as naloxone, are frequently utilized in opioid overdose cases, their use is associated with some limitations^31–35^. Moreover, the use of naloxone in some opioid poisoning, like buprenorphine, can be ineffective in clinical setting, due to the drug’s high affinity for μ-opioid receptors and long duration of action that may outlast the naloxone action^33^. Thus, the examination of alternative or complementary treatments seems justified^30^. In the present study, the lipophilicity of BPN, FTL and BTL was tested in conditions created to mimic biological systems. The drug lipophilicity was anticipated to be important in predicting the ILE-drug interaction based on the postulated mechanism of ILE’s action^4,18,19^ since drug sequestration and redistribution are partially dependent on this parameter. The experiments in this study were designed to account for potentially relevant factors that may influence the success or failure of the lipid therapy. Thus, the potential of ILE to sequester the drugs was first explored in rabbit plasma where protein binding is the major factor affecting drug distribution. After that, the same potential was investigated under more complex conditions, where disposition to the cultured cells (macrophages or fibroblasts) simulating the biophase was measured. The latter experiments were carried in protein poor medium where the distribution of the free fraction of the drug (pharmacologically active) is assessed. The collected data was used to compare the distribution of drugs and to identify the key factors that may contribute to the effective sequestration by the model ILE – Intralipid 20%.

## Material and methods

### Determination of buprenorphine, fentanyl and butorphanol concentration in plasma

The details regarding the development and validation of analytical methods for BPN and FTL have already been reported^36,37^. In brief, the HPLC-MS^2^ method for BPN was developed for a wide concentration range (0.25–2000 ng/ml) and was proved to be linear. The liquid–liquid extraction provided high method selectivity, sensitivity and recovery. The within-day repeatability and between-day reproducibility was 5.8% and 9.3%, respectively. The method LOQ was evaluated to be 0.25 ng/ml. The same detection technique was implemented for FTL, and the full validation report is presented elsewhere^37^. The method linearity was confirmed in the range of 0.02–80 ng/ml. The liquid–liquid extraction provided high sensitivity, selectivity and satisfactory recovery. The maximum observed within-day repeatability was 7.9% and between-day reproducibility was 8.7%. The matrix effect was confirmed to be < 10%. The limit of quantification (LOQ) was 0.02 ng/ml. The method was validated for two types of matrices (standard rabbit plasma and lipemic plasma) and both provided results suitable for the current application.

For BTL measurement, HPLC Waters Alliance 2695 Separation Module equipped with a Waters 2475 Multi λ Fluorescence Detector was used. The excitation wavelength was set up to 278 nm and the emission was detected at 333 nm. The chromatographic separation was conducted on Hypersil GOLD column (4.6 × 150 mm, 5 μm, Thermo Scientific, USA) with a dedicated precolumn, in temperature set to 25 °C. The mobile phase consisted of 81% 0.05 M CH_3_COONa with pH 4.0 and 19% acetonitrile, at isocratic flow rate of 1 ml/min. The sample was subjected to liquid–liquid extraction by addition of ethyl acetate:hexane (1:1 *v/v*) solution to a 500 μl of plasma sample and vortex mixing for 1 min. The upper organic layer was then transferred to the new vials, evaporated in a vacuum concentrator (Eppendorf, Germany; vacuum mode, 30 °C) and redissolved in 100 μl of methanol. No internal standard (IS) was used.

The analytical method validation was based on the construction of 9 validation curves analysed in 3 consecutive days. Analytical standard of BTL (Sigma-Aldrich, Darmstadt, Germany) was added to the blank plasma at 6 concentration levels of 1.5, 4, 10, 24, 60 and 150 ng/ml. Selectivity and specificity of the method was confirmed based on 6 blank rabbit plasma batches originated from different animals. The plasma samples were obtained from untreated experimental New Zealand rabbits housed in the Faculty of Veterinary Medicine in Wrocław and the procedure was approved by the Local Animal Experimentation Committee in Wrocław (permit number 42/2017). The calibration samples and the blank samples were subjected to the aforementioned extraction and analysis. The sensitivity of the method was based on the signal-to-noise ratio, with 3:1 ratio used for LOD and 10:1 for LOQ. For construction of validation curves, the signal was plotted against the nominal concentrations. Recovery was calculated based on the signal comparison of the BTL-loaded matrix subjected to the extraction procedure and the matrix supplemented with the drug only after the matrix extraction.

### Determination of buprenorphine, fentanyl and butorphanol concentration in cell lysates

The analysis of the three opioids of interest was conducted using Waters Alliance 2695 Separation Module connected to Waters MICROMASS QUATTRO micro API Tandem Quadrupole Mass Spectrometer (Waters, Milford Massachusetts, USA). The MassLynx 4.0 software was used for hardware control, data acquisition and processing. Analytical standards of BPN (LGC Standards, Teddington, UK), FTL (Sigma-Aldrich, Darmstadt, Germany) and BTL (Sigma-Aldrich, Darmstadt, Germany) were used for the method optimization and validation. The buprenorphine-D4 (Sigma-Aldrich, Darmstadt, Germany) was used as IS for BPN and fentanyl-D5 (Sigma-Aldrich, Darmstadt, Germany) was used as IS for FTL and BTL. Electrospray ionization was working in positive ion mode for all drugs. The capillary voltage of 1 kV was set up for BPN, and 3.5 kV was managed for FTL and BTL. Desolvation gas flow was set to 800 L/h for BPN and FTL, and 1000 L/h in case of BTL. Source temperature of 150 °C was uniformed in all the methods, and desolvation temperature was 500 °C for BPN and 350 °C for FTL and BTL. For all drugs, the stationary phase consisted of a Waters Atlantis T3 3 µm (3 × 50 mm) column, guarded with a dedicated precolumn, maintained at a constant temperature of 30 °C. Dedicated mobile phases were selected for different drugs but the flow rate was always 0.3 ml/min. For BPN solvent A was methanol and solvent B was pure water supplemented with formic acid (0.1%). The gradient started with 2% of A. From 0.75 to 0.83 min A level was raised to 98% and maintained at this level for 4 min. After 4.5 min, for the next 2 min, A concentration decreased to 2% and this level was kept for 3 min for column regeneration. For FTL, the same gradient was selected, however solvent A was acetonitrile. A different gradient was developed for BTL. Solvent A was in this case acetonitrile, and solvent B was water supplemented with formic acid. The elution started with 5% of solvent A and up to 3.33 min its concentration was raised to 100%. The pure acetonitrile flow remained until 4.67 min and after that its concentration gradually declined to 5% (until 6.67 min) and was maintained at this level until 10 min. The triple-quadrupole mass spectrometer was working in multiple reaction monitoring mode and all detection parameters are summarized in Table 1. The validation was conducted based on the calibration curves (3 curves analysed on each of 3 consecutive days).

**Table 1:** Characterisation of analytical methods developed for buprenorphine, fentanyl and butorphanol determination in cell lysates.

DRUG	Analyte	Precursor ion [m/z]	Daughter ions [m/z]	Dwell time [sec]	Cone energy [V]	Collision energy [eV]	Retention time [min]
BPN	Buprenorphine	468.3	396.3	0.3	70	55	4.8
			55.0	0.3	70	55	
	Buprenorphine – D4 (IS)	472.5	414.4	0.3	70	48	4.82
FTL	Fentanyl	337.5	188.0	0.3	31	20	5.08
			105.0	0.3	31	44	
	Fentanyl – D5 (IS)	342.0	105.0	0.3	41	44	5.1
BTL	butorphanol	328.0	310.0	0.3	45	20	4.97
	Fentanyl – D5 (IS)	342.0	105.0	0.3	41	44	5.01

The blank matrix necessary for validation was obtained by the lysis of confluent cell monolayer (J774.E and 3T3) carried out by the complete removal of the culture medium from the petri dish and subsequent addition of 2% sodium dodecyl sulphate solution. The extraction procedures were similar in all drugs and started from the addition of IS to each sample. For BPN, the buprenorphine-D4 was added at a concentration of 40 ng/ml, for FTL the fentanyl-D5 was added at a concentration of 50 ng/ml and for BTL fentanyl-D5 was added at a concentration of 60 ng/ml. During the optimization of the extraction step, special attention was paid to the necessary removal of excessive amounts of sodium dodecyl sulphate, as the matrix was very rich in this compound. To handle this issue, an additional step was introduced at the beginning of the extraction of cell lysates following the protocol developed by Zhou et al.^38^. Precipitation of potassium dodecyl sulphate was obtained by the addition of 4 M potassium chloride to the 300 µl (in case of BPN and FTL) or 100 µl (in case of BTL) of the sample. After that, the sample was centrifuged and only the clear sediment-free fraction was subjected to further liquid–liquid extraction. Fife hundred µl of 25% ammonia water solution (v/v) (Stanlab, Lublin, Poland) was added to each sample to facilitate the migration of the drug to the organic solvents. For BPN and FTL the extraction solution composed of 1-chlorobutyl:acetonitrile solution (4:1 v/v), but in the case of BTL ethyl acetate:hexane (1:1 v/v) was used. The extraction was only 1 min vortex mixing for FTL and BTL, whereas for BPN it was prolonged to 20 min. After that, the clear upper layer was collected and evaporated until dryness in a vacuum concentrator (Eppendorf, Hamburg, Germany; vacuum mode, 30 °C). The residues were redissolved in 100 µl of methanol.

### Evaluation of the distribution coefficient in biological setting

The typical parameter used to characterise the lipophilicity of the substance is the partition coefficient measured between octanol and water^39^. In this study a related parameter, the distribution coefficient, was measured. This parameter, in contrast to the partition coefficient, is measured in biologically relevant conditions and in the range of physiological pH values where the degree of drug’s ionisation reflects the situation in vivo^40^. To evaluate this parameter, we used normal fresh rabbit plasma obtained as describe earlier. Plasma was in vitro supplemented with 2% addition (v/v) of Intralipid 20% (Fresenius Kabi, Uppsala, Sweden) and then spiked with 250 ng/ml and 500 ng/ml of FTL, BPN and BTL. Intralipid 20% is a commercially available lipid emulsion consisting of soybean oil, egg yolk phospholipids, glycerin and water^41^. The final Intralipid concentration to which cells were exposed was based on the expected concentration in human subjects obtained after the recommended bolus emulsion administration during lipid rescue therapy (1.5 ml/kg) and the amount evaluated by French et al.^20^ to produce adequate drug sequestration under in vitro conditions. Since the opioid poisoning cases may vary significantly in terms of the dose, we selected the concentrations of the drugs that fairly exceed their therapeutic ranges and in clinical conditions would undoubtedly produce severe toxicity. The same analytical standards as during the method validation were used for BPN (LGC Standards, Teddington, UK) and FTL (Sigma-Aldrich, Darmstadt, Germany), but in the case of BTL, pharmaceutical grade standard kindly provided by Richter Pharma AG (Wels, Austria) was utilized. After spiking with respective drugs, all plasma samples were mixed on a horizontal shaker and incubated in 37 °C for 30 min. After that, each sample was divided into two parts: one was analysed according to standard procedures, and the second was ultracentrifuged first. The ultracentrifugation was performed in 133 000 g for 20 min (Optima L‐90 K ultracentrifuge, Beckman Coulter). The bottom lipid-sparse layer was carefully collected using syringe and needle, and each tube was punctured from the bottom. The clear plasma was slowly and carefully collected to ensure that no lipids were collected alongside. The concentration of each drug was evaluated according to the procedures as described in the earlier sections.

Relative change in plasma concentration was calculated according to the equation:

$$\% Lip= \frac{{C}_{whole}-{C}_{centr}}{{C}_{whole}} \times 100\%$$

where %Lip is a percent of a drug that was located in the lipid layer, C_whole_ is a concentration of a drug that was observed in a homogenous sample before centrifugation, C_centr_ is a concentration of a drug that was measured in a separated clear plasma after centrifugation. The percentage of the drug in the clear lipid free plasma was calculated by simple subtraction *%Plasma* = *100%—%Lip*. The distribution coefficient (K_d_) was calculated as a *%Lip/%Plasma* ratio. The experiment was performed in three independent repetitions.

The differences between the tested concentrations (250 ng/ml and 500 ng/ml) of the same drugs were tested by Mann–Whitney–Wilcoxon test. Kruskal–Wallis test was conducted to assess potential differences between the opioids. Dunn’s test was used as a post hoc test. *p* < 0.05 was considered as indication of an important difference in all tests. All statistical calculations performed in the present study were conducted using R (version 4.0.3, The R Foundation, Vienna, Austria) and RStudio (version 4.1.0, RStudio, Boston, MA, USA) software.

### Disposition of the drugs to the cell monolayer

To simulate tissue drug disposition, the transfer of three opioids to the biophase was investigated in vitro. J774.E murine macrophages and 3T3-Swiss albino murine fibroblasts cell lines were used as the biophase model. 3T3 cell lines was bought from the American Type Culture Collection (ATCC, Rockville, MD, USA) and the J774.E cell line was obtained from Hirszfeld Institute of Immunology and Experimental Therapy - Polish Academy of Sciences (Wrocław, Poland). The two lines were selected based on their physiological features. The J774 macrophages are an established model for the process of chylomicron remnants uptake^42–45^. These particles are very similar to the lipid droplets found in Intralipid^46^ and J774 macrophages are known to internalise triglycerides from Intralipid^47^. On the other hand, the 3T3 fibroblasts are of much lesser importance in terms of lipids metabolism and are not as efficient in lipid capture^42^. Therefore, the use of these two cell lines allowed for the determination of the possible role of active lipid droplet uptake (presumably by phagocytosis) in the potential modification of the distribution of lipid-bound fraction of opioids.

In this experiment, 4 × 10^6^ and 1.5 × 10^6^ cells were seeded on petri dishes (TPP, Switzerland) for J774.E and 3T3, respectively. The number of cells was selected to ensure conditions close to confluent culture. All experiments involving cell cultures were carried out using the same standard media composed of RPMI-1640 solution (Institute of Immunology and Experimental Therapy, Wrocław, Poland) with 10% addition of foetal bovine serum (Gibco, USA), L-glutamine (Sigma, United Kingdom) and antibiotics (penicillin and streptomycin, Sigma, Germany). During the experiment the cells were frequently controlled by light microscopy to ensure the adequate cells’ appearance, vitality, and their ability to multiplicate. The cells were incubated overnight at 37 °C in a humidified atmosphere of 5% CO_2_. After that, to each pair of plates FTL, BPN or BTL was added to the culture medium to obtain the concentrations of 100, 250 and 500 ng/ml. All plates were incubated on horizontal shaker (37 °C, 5% CO_2_) for 10 min to allow free drug distribution to the cells. Then, Intralipid 20% was added to one dish of each pair to obtain a concentration of 2% (*v/v*) and an equal volume of culture medium was added to the control dishes (in all cases the total volume of final incubation solution was 10 ml). The dishes were stirred and incubated under the same conditions as before for further 4 h. After that time, all medium was carefully removed. One ml of 2% sodium dodecyl sulphate was then added to the dishes and they were incubated overnight for a complete lysis of the cells. All lysates were subjected to the drug concentration evaluation to determine the drug concentration in the cell monolayer. The experiment was conducted in at least three independent repetitions for each drug. After the evaluation of the results, an additional repetition for BPN at the concentration of 250 ng/ml and 500 ng/ml was performed using 3T3 cell line. This additional repetition was conducted to ensure that the very large differences between this drug and other tested opioids did not result from human or analytical mistakes. The cells were additionally observed under the inverted optical microscope (Zeiss, Primo Vert, Germany), to detect any signs of morphological changes (i.e. opaque appearance due to lipid droplet uptake into the cytoplasm).

The Mann–Whitney–Wilcoxon test was used to explore the differences between the two cell lines. The Kruskal–Wallis test followed by Dunn’s test was used to assess potential differences of distribution coefficient between the tested opioids and between the different concentrations of the same drug. *p* < 0.05 was considered as an indication of important difference in all tests.

### Evaluation of Intralipid cytotoxicity

To exclude the possibility of toxic effects of high concentrations of Intralipid 20%, viability test on J774.E and 3T3 cells by means of the MTT assay was performed. The procedure for both lines was the same. Cells were seeded in 96-well plates (TPP, Switzerland) at the concentrations of 10^4^ cells per well for J774.E and 3 × 10^3^ cells per well for 3T3 line. They were incubated overnight in standard medium at 37 °C in a humidified atmosphere of 5% CO_2_. Then, aliquots of Intralipid 20% concentrations prepared in cell medium were added to obtain 0.0625%, 0.125%, 0.25%, 0.5%, 1%, 2%, 4% and 8% concentration of the final commercial emulsion (v/v). After 48 h incubation (37 °C, 5% CO_2_), medium was exchanged for a fresh one and MTT assay was carried out^48^. The test is based on the enzymatic reduction of the tetrazolium salt MTT (3-(4,5-dimethylthiazol-2-yl)-2,5-diphenyl-tetrazolium bromide) in living, metabolically active cells. After MTT addition, cells were incubated for 4 h and lysis buffer was added. The metabolite, purple-colored formazan, was measured colorimetrically after 24 h. The absorbance was evaluated using a spectrophotometric microplate reader (Tecan Spark 10 M, Switzerland) at a wavelength of 570 nm (reference 630 nm). The absorbance of control cells was taken as 100%. Cell viability was determined as follows: % viability = (mean absorbance in the test wells/mean absorbance for control wells) × 100. During the experiment the cells were frequently looked at by means of light microscopy to ensure the adequate cells’ phenotype and proliferation. The results were obtained from 3 independent experiments.

### Plasma protein binding

The ultrafiltration method was used to evaluate the free fraction and protein binding of BPN and FTL in the rabbit plasma. The plasma was originated from the same pool as described in 2.3. *Evaluation of the distribution coefficient in biological setting* section. Centrifugal devices (Nanosep Omega 10 k, Pall Corporation, Puerto Rico) were pre-treated with Tween 20 to reduce the nonspecific binding to the membranes^49^. Then, 500 µl of fresh rabbit plasma, spiked with 5, 20 or 100 ng/ml of BPN or FTL, were inserted to the devices and incubated with constant mixing at 37 °C for 1 h. After that time, the samples were centrifuged in 1000 g for 10 min and then in 2000 g for 20 min and the filtered fraction was subjected to the drug analysis alongside with the initial plasma sample. The extent of the non-specific binding to the membrane was additionally investigated using the same drug concentrations and the same experimental protocol but phosphate-buffered saline was used as matrix instead of plasma. The drug free fraction and protein binding were calculated using following equations:

$${f}_{u}=\frac{{C}_{FP}}{(1-NSB)\times {C}_{TP}}$$

$$\%PB=100\times (1-{f}_{u})$$

where *f*_*u*_ is fraction unbound, *C*_*FP*_ is free plasma concentration, *C*_*TP*_ is total plasma concentration, *NSB* is non-specific binding and *%PB* is protein binding^50^. Each experiment was performed in three independent repetitions.

### Comparison of the measured in biologically relevant conditions distribution coefficient to the logP values

The Kendall rank correlation coefficient test was used to compare the obtained results of the drugs lipophilicity with the values of octanol:water partition coefficient obtained from the published scientific literature^27–29^. The value of *p* < 0.05 was considered as an indication of statistical significance. The distribution coefficient obtained after ultracentrifugation and cell culture distribution was compared to each other for each drug using Mann–Whitney U test.

## Results

### Analytical methods for buprenorphine, fentanyl and butorphanol in rabbit plasma

The results regarding the validation of analytical methods for BPN and FTL in rabbit plasma have already been reported elsewhere^36,37^. The analytical method for BTL was developed purposely for this study. It is characterized by very good selectivity as confirmed by the inspection of blank samples. The method was linear (R^2^ = 0.9999) in the range of all concentrations observed in this study (1.5–150 ng/ml). The intra-day coefficient of variation was 10.72%, whereas between-day coefficient of variation was 19.05% for the concentration of 1.5 ng/ml. The recovery of the extraction ranged from 71 to 82%. Based on signal to noise ratio, limit of detection (LOD) and LOQ were calculated to be 0.16 and 0.54 ng/ml, respectively. All elaborated and validated analytical methods were concluded to be suitable for the purpose of this study. The obtained results were within the acceptance criteria consistent with the European Medicines Agency guidelines^51^. The range of the methods was always adequate to the concentrations found in a given matrix.

### Analytical methods for buprenorphine, fentanyl and butorphanol in cell lysates

The methods, elaborated and validated separately for each drug, resulted in very good sensitivity, selectivity, range, linearity, recovery, precision and accuracy. All these parameters are summarised in Table 2. The methods were concluded to be suitable for further application in the evaluation of drug distribution to the biophase.

**Table 2:** Validation parameters obtained for three opioids in cell lysates.

Parameter	Buprenorphine	Fentanyl	Butorphanol
LOD [ng/ml]	0.3	0.3	1
LOQ [ng/ml]	1	1	5
matrix effect	9.75 ± 8.57%	9.45 ± 3.64%	0.23 ± 4.68%
concentration range [ng/ml]	1–800	1–400	1–300
determination coefficient (R^2^)	0.9994	0.9999	0.9996
calibration curve	y = 2.1137x	y = 0.1632x	y = 0.0134x + 0.0126
recovery	82.7–101.7%	96.1–101.8%	110.1–121.1%
within day variability	6.5–11.0%	3.7–17.5%	1.0–10.5%
between day variability	6.5–11.7%	6.7–16.9%	4.7–9.8%

LOD—limit of detection, LOQ—limit of quantification. The results were obtained from 9 validation curves, except for the within day variability where 3 curves were considered.

### Evaluation of the distribution coefficient in rabbit plasma

The ultracentrifugation of the rabbit plasma samples resulted in efficient separation of the upper lipid layer from the clear aqueous fraction of plasma. The percentage of drug distributed to ILE-free plasma and ILE layer can be appreciated in Table 3.

**Table 3:** In vitro disposition of the investigated opioids, between aqueous plasma and lipid layer.

Drug		buprenorphine	fentanyl	butorphanol
Concentration 250 ng/ml	%Plasma	65.21–65.85^*a*^	71.47–83.39^*ab*^	87.97–93.99^*b*^
	%Lip	34.15–34.79^*a*^	16.61–28.53^*ab*^	6.01–12.03^*b*^
	K_d_	0.53 ± 0.01^*a*^	0.31 ± 0.10^*ab*^	0.10 ± 0.04^*b*^
Concentration 500 ng/ml	%Plasma	61.83–65.26	71.77–88.34	84.46–86.45
	%Lip	34.74–38.17	11.66–28.23	13.55–15.54
	K_d_	0.58 ± 0.04	0.22 ± 0.15	0.17 ± 0.02

%Plasma—percentage of the drug that was found in lipid-free plasma, %Lip—percentage of the drug that was found in the upper lipid layer, K_d_—distribution coefficient evaluated in rabbit plasma and lipids originated from Intralipid, n = 3, values that do not share a superscript letter at the same row are considered statistically different, lack of superscript latter indicates lack of statistical difference. No differences were found between tested concentrations for the same drugs.

The statistical analysis indicated that the distribution coefficient was not influenced by the concentration of the tested drug. The only difference between the opioids was found between BPN and BTL for concentration 250 ng/ml (*p* = 0.0036).

### Disposition of the drugs to the cell monolayer

**Figure 1:**
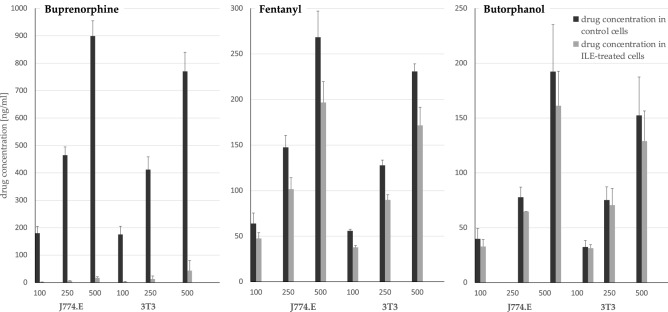
Concentrations of different drugs in cells incubated with or without Intralipid. Each drug was tested at three different concentrations in two cell lines, as denoted on the horizontal axes. Black columns show concentrations in cell monolayer incubated with the drugs and without Intralipid, and light grey columns show the concentrations after incubation with Intralipid. Vertical bars represent standard deviation.

Figure 1 shows the differences in drug concentrations evaluated in the cell monolayer incubated with and without Intralipid 20%. Although each drug was administered to the petri dish at the same concentrations (to obtain the final concentration in medium of 100, 250 and 500 ng/ml), sharp differences were revealed between the three drugs. The most prominent change can be seen for BPN. Drug concentrations in the control cells (incubated without Intralipid) were very high in both tested cell lines indicating very intensive BPN distribution to the cells as compared to the other drugs. However, the addition of Intralipid led to a shift and intracellular BPN concentration plummeted, suggesting that a significant fraction of the drug was preferentially distributed to the lipid droplets in the medium.

This enormous difference is also reflected in the values presented in Table 4. The difference between the two incubations (with or without Intralipid) is expressed as percent change, and it is visible that, compared to the other tested opioids, BPN showed the most pronounced ratio change. The comparison of distribution coefficients revealed significant difference between the values for BPN and BTL in all tested concentrations in both cell lines.

**Table 4:** The ratio of drug concentration evaluated in the cells in Intralipid positive group and Intralipid free group (n = 3), as well as percent change in the concentration caused by the Intralipid addition. The results are presented as mean ± standard deviation.

DRUG	buprenorphine	fentanyl	butorphanol
cell line		J774.E	
Concentration in medium [ng/ml]	Ratio of drug concentration		
100	0.011 ± 0.001^*a*^	0.749 ± 0.044^*ab*^	0.833 ± 0.055^*b*^
250	0.013 ± 0.002^*a*^	0.688 ± 0.033^*ab*^	0.838 ± 0.100^*b*^
500	0.019 ± 0.005^*a*^	0.733 ± 0.032^*ab*^	0.841 ± 0.026^*b*^
	Percent change in concentration		
100	98.91 ± 0.15%^*a*^	25.11 ± 4.39%^*ab*^	16.67 ± 5.46%^*b*^
250	98.71 ± 0.24%^*a*^	31.21 ± 3.30%^*ab*^	16.20 ± 10.01%^*b*^
500	98.12 ± 0.50%^*a*^	26.72 ± 3.19%^*ab*^	15.87 ± 2.55%^*b*^
Cell line		3T3	
concentration in medium [ng/ml]	ratio of drug concentration		
100	0.015 ± 0.015^*a*^	0.676 ± 0.016^*ab*^	0.970 ± 0.058^*b*^
250	0.034 ± 0.031^*a*^	0.705 ± 0.075^*ab*^	0.933 ± 0.074^*b*^
500	0.058 ± 0.054^*a*^	0.746 ± 0.113^*ab*^	0.849 ± 0.016^*b*^
	Percent change in concentration		
100	98.45 ± 1.53%^*a*^	32.40 ± 1.59%^*ab*^	2.99 ± 5.83%^*b*^
250	96.65 ± 3.11%^*a*^	29.53 ± 7.51%^*ab*^	6.65 ± 7.41%^*b*^
500	94.18 ± 5.42%^*a*^	25.42 ± 11.29%^*ab*^	15.12 ± 1.58%^*b*^

Values in a row that do not share a superscript letter are considered statistically different (*p* < 0.05). No differences were found between tested concentrations and cell lines used within the same drug for every tested opioid.

A different situation was observed in the case of FTN and BTL. The concentrations of both drugs in the control cells were much lower than in the case of BPN, and the impact of Intralipid was not as pronounced. In the case of both drugs, the ratio of change in the intracellular drug concentration was independent of the cell type and the drug concentration.

**Figure 2:**
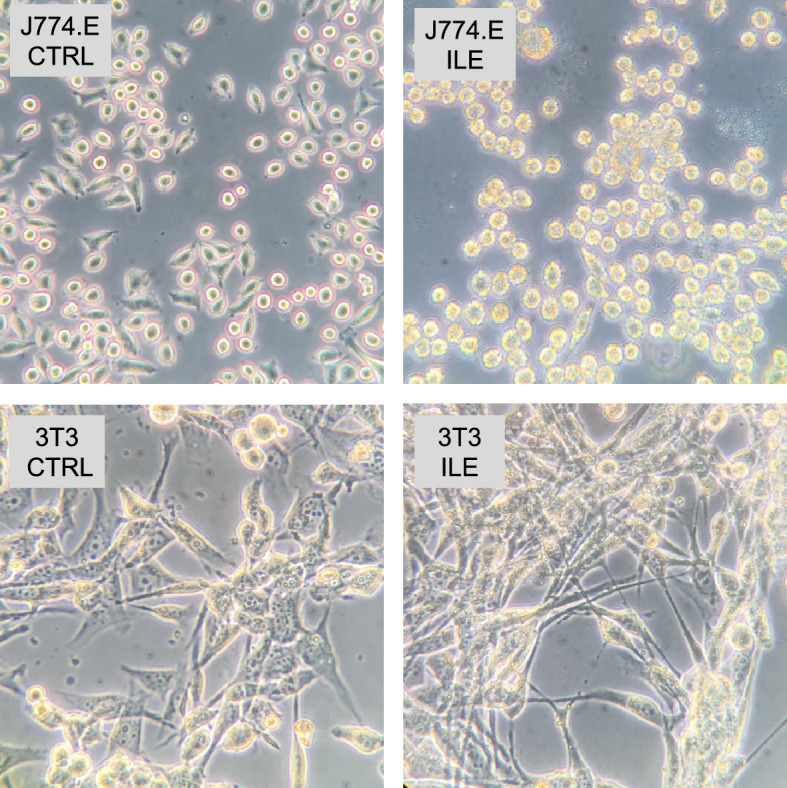
J774.E and 3T3 cell lines after the exposure to Intralipid (8%) (ILE), in comparison to the cells cultured in standard conditions (CTRL).

The distribution of lipid droplets was additionally investigated optically with the inverted optic microscope and revealed that the cytoplasm of J774.E cells was more granular and opaque after the incubation with Intralipid. In 3T3 cells, this effect was much less pronounced, as can be seen in Fig. 2.

**Figure 3:**
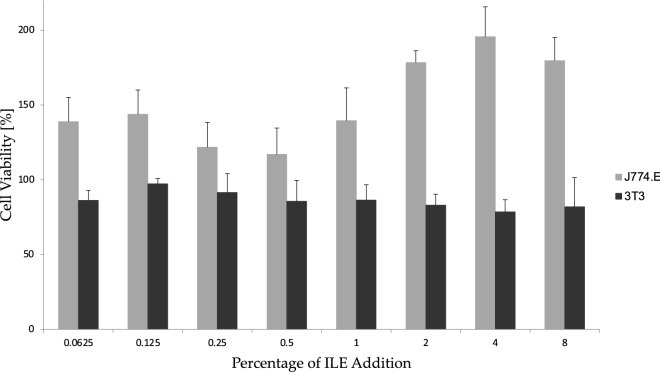
The impact of Intralipid on the viability of two cell lines. The values are presented as mean viability compared to the control cells, and vertical bars indicate the standard deviation associated with the result (n = 3).

The measured cell viability in both cell lines exposed to ILE is depicted in Fig. 3. The two cell lines presented a very different pattern of response in the MTT assay. Fibroblasts (3T3-Swiss albino) showed a stable level of metabolic activity, oscillating around 100% (baseline of the control cells) regardless of the concentration of ILE. A different trend was observed in the macrophage line (J774.E). At higher concentrations, ILE stimulated the metabolic activity of cells which resulted in an almost two-fold increase in response at the concentrations exceeding 2% of ILE as compared to the untreated control. Even the smaller addition of ILE resulted in the increased metabolic activity in comparison to the control cells. Nevertheless, no apparent viability inhibition was observed in either cell line.

### Plasma protein binding

Despite the fact that during the procedure all efforts were made to prevent the nonspecific drug binding, in the case of BPN the results revealed that more than 99% of the drug was bound to the filtration membrane. Thus, credible calculation of protein binding for BPN was not possible. In case of FTL, the maximal calculated non-specific binding was 34.89%, thus evaluation of protein binding was possible. The values of fraction unbound and protein binding, corrected for the non-specific binding, are gathered in Table 5. The assay showed that regardless of FTL concentration, the protein binding was on a stable level of 80–85%, indicating that only relatively small fraction of the drug can be found in the plasma as a free drug.

**Table 5:** The fraction unbound (*f*_*u*_) and protein binding (*%PB*) of fentanyl in rabbit plasma (n = 3). The results are presented as mean ± SD values.

FTL concentration [ng/ml]	5	20	100
*f*_*u*_	0.18 ± 0.02	0.17 ± 0.02	0.16 ± 0.01
*%PB*	82.34 ± 1.93	82.58 ± 1.95	84.44 ± 0.89

### Experimentally evaluated distribution coefficient vs. logP

**Figure 4:**
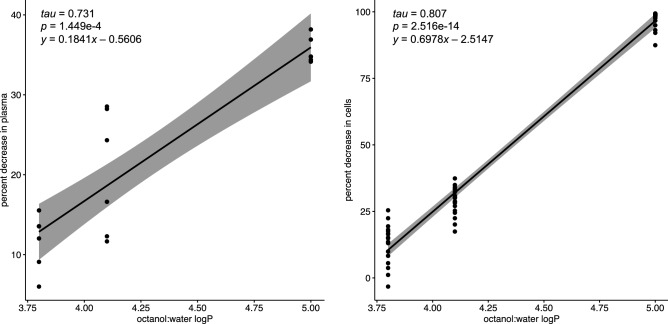
Partition coefficient values of three tested opioids plotted against the decrease in drug concentration resulting from the addition of 2% Intralipid (*v/v*). Two different media were explored: fresh rabbit plasma (left panel, n = 6), and cell culture (right panel, n = 18). For each relationship the Kendall rank correlation coefficient was calculated (tau), and the equation for the regression line was provided. As indicated by *p* < 0.05, in both cases the correlation was significant.

In the present work, one of the objectives was to investigate whether a classically evaluated partition coefficient between octanol and water is a good predictor of drug sequestering potential of ILE under biological conditions. To assess that, the results of the two aforementioned experiments were compared with the logP of the drugs. The distribution coefficient evaluation in plasma revealed the same tendency as logP—BPN is the most lipophilic among the drugs used in this study (mean distribution coefficient of 0.552), FTL is distinctly less lipophilic (mean distribution coefficient of 0.264), and BTL is the least lipophilic from the three opioids (mean distribution coefficient of 0.133). These results are well in agreement with the measurements obtained in standard octanol:water partitioning approach (logP of 5.0, 4.1 and 3.8 for BPN, FTL and BTL, respectively)^27–29^. Kendall rank correlation coefficient test demonstrated high correlation of these results (Fig. 4). The more complex investigation of the drug distribution in the cellular model also resulted in a significant correlation with the octanol:water partitioning coefficient (Fig. 4). In this case, the correlation was even stronger, with *tau* = 0.807.

The comparison between the two models (cell-free plasma and monolayer cell culture) revealed that the method of testing did not have an impact on the results obtained in the case of BTL (*p* = 0.4537). However, for BPN and FTL important differences were noted depending on the selected method of the disposition coefficient measurement (*p* = 1.8 × 10^–9^ and *p* = 1.5 × 10^–2^ for BPN and FTL, respectively).

## Discussion

Poisonings with synthetic opioids are one of the major concerns for many health agencies^52^. Their substantial potency as well as high availability from both legal and illegal sources cause significant impact on public health including societal and economic adverse effects^52^. Considering this significant impact, searching for measures to address the issue at different levels is vital. One countermeasure that can be offered is fast and effective treatment of poisoned individuals. In recent years several new strategies have been developed to extend possible treatment options beyond the standard administration of the opioid antagonist, naloxone. Intranasal administration of nalmefen, another opioid receptor antagonist, offers higher affinity, rapid onset and longer duration of action^53^. Another novel drug, methocinnamox, may provide not only reversal of toxicity but also protects against opioid overdose^54^. Naloxone stabilized by nanoparticles and serotonin used as a respiratory stimulant have also been reported^55^. Other options include the so-called biomimetic “nanosponges” that, after intravenous administration, can prevent migration of opioids into the central nervous system^56^. Several antibody-based strategies were proposed as well ^56^ and, recently, cyclodextrin scaffolds have been proposed to specifically bind fentanyl in blood^57^. However, some of these strategies have never left the laboratory setting.

In our work we decided to test a drug with an established position on the market—ILE. The relative simplicity of the solution and high availability of this product should be considered as an advantage of this approach. Moreover, its efficacy has been confirmed in some other resuscitation protocols.

In literature, the assessment of ILE rescue therapy effectiveness is predominantly based on in vivo and clinical studies^58–60^. Nevertheless, there are many in vitro models that may deliver useful mechanistic insights and may allow predictions of ILE efficacy regarding interactions with different molecules^22,26,61,62^. In the present study, two different approaches were used to test ILE’s capability to sequester selected opioids and measure possible “lipid sink” effect. First, we employed ultracentrifugation technique to destabilize the plasma-emulsion system and evaluate how concentration of opioids changed during the process. Secondly, the drug removal from cell monolayer (sequestration in the medium) was investigated. The former experimental setup was inspired by French et al.^20^. In the cited experimental work, they investigated the distribution coefficient of eleven miscellaneous drugs using human serum and Intralipid with a protocol identical to the present study. The authors observed a strong correlation between the octanol:water partitioning coefficient and the distribution coefficient of drugs measured in plasma. Moreover, to a lesser extent, the drugs’ volume of distribution was also correlated with the measured distribution coefficient. They did not include any opioid drug in their experiments but, based on the developed model, they predicted that 2% (*v/v*) addition of Intralipid 20% should decrease FTL serum concentration by 35%. This value is not far from the results of the currently presented experiment (Table 2). Unfortunately, no predictions for BPN and BTL were provided.

In contrast to the ultracentrifugation test, the cell culture model of drug distribution to the biophase developed in the present work is, to our best knowledge, rather unique in its application to the studies on lipid emulsions. Other models using isolated cells or tissues aimed rather at the assessment of the pharmacodynamic efficacy of the ILE treatment under ex vivo conditions^63–66^. The advantages of the model described in the current study are relative simplicity and the ability to deliver key information about the sequestration potential of ILE for a given drug. In this simple system consisting of cell monolayer (the biophase), medium and Intralipid, two cell lines with different physiology and morphology were tested. Despite the initial expectations, no important differences were found between the cell types (Table 4). Since J774.E macrophages are known to efficiently accumulate chylomicrons^43^, it was expected that they could serve as a viable model for the “lipid shuttle” theory^4^ where the cellular uptake of lipid droplets may translate at a larger scale to the increased clearance of the drug entrapped in these droplets. However, the results suggest that this process may be of limited importance or at least has a limited impact on the intracellular drug concentrations. Simple diffusion through the cell membrane was likely far more important than the possible effects related to the active uptake of opioid-rich lipid droplets suggesting that the “lipid sink” mechanisms rather than the “lipid shuttle” is operative in the current model. Moreover, possible cytotoxic impact on the cell lines was also excluded (Fig. 3) and changes in opioids’ concentrations observed in the disposition study could not be attributed to the decrease in cell viability and loss of integrity.

In the tested environment, all three drugs exhibited different sequestration potential by the lipids, but BPN was by far the most efficiently bound one (Fig. 1, Table 4). The drug was so efficiently redistributed from the cell monolayer, that additional repetition of the experiment was conducted to verify this effect. Despite moderate differences in the logP values (5.0, 4.1 and 3.8 for BPN, FTL and BTL, respectively^27–29^) the sequestration of BPN by lipids, regardless of its concentration and cell line used, was extremely efficient. The origin of this intriguing effect cannot be entirely elucidated based on the conducted experiments, however, the very nature of the logP can offer a possible explanation. Namely, logP is a parameter calculated based on the log-transformed ratio of the compound’s concentrations measured in two phases^23^. Thus, even a small apparent change in the logP value can translate to a major change in drug distribution when measured in a linear scale, as it is in the case of the current study.

The two in vitro models applied in this work for the assessment of lipophilicity and the prediction of tissue distribution (plasma ultracentrifugation and the biophase distribution) provided different results. The experiments indicated that the lipid-bound fraction in plasma (plasma ultracentrifugation test) is lower than the ILE-induced decrease in the drug concentration in the cell monolayer (the biophase model) in case of BPN and FTL (Tables 3 and 4). For BPN, around 36% of the plasma drug was sequestrated in ILE lipids whereas the drug concentration in cells dropped by as much as 97% after the addition of Intralipid to the medium (at the same concentration as in plasma). In contrast, for FTL approx. 20% was sequestered in the ILE lipids in plasma which corresponded to 29% drop in cell drug concentration. For BTL 12% was sequestered in the ILE lipids in plasma and the addition of ILE led to the decrease in cell drug concentration of only 13%. This may suggest that, especially for BPN, even relatively small drop in the plasma disposition coefficient does not exclude an important impact of ILE on drug penetration into cells.

These discrepancies in the results of the two tested approaches can be caused by many factors like drug metabolism, degradation, and also plasma protein binding. The analytical methods applied in this study quantify the total drug concentration, without distinguishing between the protein-bound fraction and the free drug. However, some authors argue that only the free drug can be efficiently sequestered in the ILE droplets^18,67^. Thus, the more the drug is bound to the proteins, the less of it will be available for transition into the lipid fraction^68^. What is fundamentally important in this context is the fact that only the free fraction is both, pharmacologically/toxicologically active and available for elimination. The free and the bound fraction (whether protein-bound or entrapped in lipid droplets) are in dynamic equilibrium, therefore any change in the relevant conditions (e.g. increase in the affinity of binding or in the volume of the reservoir for binding) will immediately affect the latter fraction. According to the “lipid sink” theory, by using intravenous emulsions in the patient we simply aim at increasing the inactive fraction (bound to both proteins and lipids in plasma). However, using the full plasma (acellular) system we cannot distinguish fractions bound to proteins and lipids. Furthermore, the higher the protein-bound fraction of the drug, the more biased are the measurements of the disposition coefficient. The protein binding data for opioids seem to explain the results of the present study. More than 90% of BPN is bound to plasma albumin, gamma-globulin and alpha-acid glycoprotein^69–71^. Thus, the small free fraction available for lipid sequestration may be responsible for the relatively low value of disposition coefficient obtained in the ultracentrifugation test. The two other opioids bind to plasma proteins to a lesser extent [around 80% for FTL as evaluated in this study is bound to proteins and similar values were reported for BTL in literature^72^], thus, lipid sequestration in plasma should be less biased than in the case of BPN. All these considerations lose relevance, however, when the drugs are tested in the cell culture conditions. The complete culture medium contains only 10% foetal bovine serum so plasma proteins are at least 10 times diluted as compared to full plasma used in the first experiment. Since the protein concentration can have an impact on the free drug concentration^73^ it is possible that under these conditions the majority of the drug remains in the free form. This may explain why BPN could be so efficiently sequestered in the medium and later washed away from the biophase. However, in plasma, the sequestration is likely to be hampered by a strong drug interaction with the abundant proteins. Combining the information from these two systems (plasma and cell culture) provides us with the insight into the relations between the biologically relevant fractions and allows better predictions for in vivo scenarios. E.g. if the free drug is avidly bound by lipid droplets but is also showing high protein binding (BPN case), we can conclude that despite high lipophilicity, the clinical benefit may be limited as the available protein-unbound fraction is very small and we cannot add much drug immobilization with the emulsion. This hypothesis should be confirmed by additional evaluation of distribution coefficient under biological conditions when only the free fraction is measured. Unfortunately, such tests are problematic for BPN as it binds to many types of membranes used for these studies. This issue was reported in the literature^69^ and was observed also during the tests conducted in our laboratory.

The use of diluted proteins in cell culture medium can be seen as biologically less relevant as compared to plasma or serum. Nevertheless, as the ILE addition can sequester only the free fraction of the drug^18,67^, the investigation of the emulsion’s impact on the unbound fraction in cell culture medium is, paradoxically, of superior value as compared to plasma measurements which are further complicated by plasma proteins. Taking this into account, the disposition to the biophase seems to provide less biased predictions of the ILE efficacy in the drug sequestration, as this model accounts for two crucial factors: the impact on the free fraction exclusively and the drug penetration to the cells. However, this hypothesis should be confirmed in conditions where only the free drug concentrations would be measured.

It may be argued that the experimental setting would be more complete if the cellular arm of our study would have additional incubation of cells in full rabbit plasma. However, most cells do not tolerate well high concentrations of plasma/serum and their physiology would have probably been severely affected. Adding bovine serum albumin to the medium to increase total protein concentration would also not make the setting more relevant as opioids bind to other, less abundant protein fractions^71,74^. Although the lack of such additional comparison in the cellular experiments may be perceived as a limitation, we believe that the aforementioned factors significantly limit its feasibility.

A possible limitation of the study is that the concentration of metabolites was not measured and the metabolism capacity of the selected cell lines is not well known. The activity of cytochrome P450-3A4, involved in the metabolism of opioids^75^, is very unlikely in the 3T3 fibroblasts^76^ but in J774.E macrophages it cannot be entirely excluded^77^. Nevertheless, the study design applied in this research is expected to account for any possible residual metabolism that should be similar in the ILE-treated and control culture dishes. The MTT test showed that ILE had little to no impact on the cells’ viability, but its impact on the cytochrome P450 remains unknown. Thus, the possibility of the different metabolic rates may also be considered as a potential source of variability in the two in vitro models.

Despite all these discrepancies, the results of the cell-free studies using plasma as well as the results of the in vitro biophase model correlated well with the LogP values for the opioids under investigation suggesting a good predictive value of these tools for the assessment of the drug:ILE interaction (Fig. 4). However, the relation was far from a simple y = x function. It should be kept in mind that physicochemical drug properties (as partition coefficient) are indeed very important in the modulation of interaction with ILE, but other valid factors should not be overlooked in the attempt of translating these properties to the biological conditions. One of these crucial factors revealed in the present investigation is protein binding. Thus, while predicting the possible benefits of the use of ILE in the modulation of toxicant/drug disposition under clinical conditions, the partitioning coefficient evaluated in octanol:water experiments should be used with caution, as it does not account for many factors present in the biological conditions like distribution to different cells and tissues and interactions with other components of body fluids.

Findings presented in this work cannot replace the in vivo studies investigating interactions between ILE and opioids during the poisoning. However, the present work provides valuable predictions of the potential susceptibility of different opioids to sequestration in lipids and provides an alternative approach for the evaluation of biologically relevant relation between drug lipophilicity and distribution to the components of the biological milieu. Therefore, the proposed approach may contribute to future design of efficient therapeutic protocols utilizing the potential of ILE to modulate pharmaco- or toxicokinetic scenarios under clinical conditions. Moreover, it may easily be applied other classes of drugs and xenobiotics.

## Data Availability

The datasets generated and/or analysed during the current study are available in the Zenodo repository, https://zenodo.org/record/6091872#.YgvMky8w0cg.
